# Role of the Human Breast Milk-Associated Microbiota on the Newborns’ Immune System: A Mini Review

**DOI:** 10.3389/fmicb.2017.02100

**Published:** 2017-10-25

**Authors:** Marco Toscano, Roberta De Grandi, Enzo Grossi, Lorenzo Drago

**Affiliations:** ^1^Laboratory of Clinical Microbiology, Department of Biomedical Sciences for Health, University of Milan, Milan, Italy; ^2^Villa Santa Maria Institute, Como, Italy; ^3^Clinical-Chemistry and Microbiology Lab, IRCCS Galeazzi Orthopedic Institute, University of Milan, Milan, Italy

**Keywords:** human milk, milk microbiota, colostrum, immunomodulation, newborn’s immune system, AutoCM

## Abstract

The human milk is fundamental for a correct development of newborns, as it is a source not only of vitamins and nutrients, but also of commensal bacteria. The microbiota associated to the human breast milk contributes to create the “initial” intestinal microbiota of infants, having also a pivotal role in modulating and influencing the newborns’ immune system. Indeed, the transient gut microbiota is responsible for the initial change from an intrauterine Th2 prevailing response to a Th1/Th2 balanced one. Bacteria located in both colostrum and mature milk can stimulate the anti-inflammatory response, by stimulating the production of specific cytokines, reducing the risk of developing a broad range of inflammatory diseases and preventing the expression of immune-mediated pathologies, such as asthma and atopic dermatitis. The aim of the present Mini Review is to elucidate the specific immunologic role of the human milk-associated microbiota and its impact on the newborn’s health and life, highlighting the importance to properly study the biological interactions in a bacterial population and between the microbiota and the host. The Auto Contractive Map, for instance, is a promising analytical methodology based on artificial neural network that can elucidate the specific role of bacteria contained in the breast milk in modulating the infants’ immunological response.

## Introduction

The human milk is a rich and complete nourishment that is essential for the correct development of the infant’s organism (Ballard and Morrow, 2013).

The first milk produced by mothers after the delivery is called colostrum and it is biochemically and functionally different from the mature milk (Castellote et al., 2011). Colostrum, indeed, contains high concentration of lactoferrin, Immunoglobulin A (IgA), leukocytes and specific developmental factors, and a low amount of lactose, potassium and calcium, underlying its immunological functions rather than nutritional (Kulski and Hartmann, 1981; Pang and Hartmann, 2007).

From 5 days to 2 weeks postpartum, there is the production of transitional milk which shares some characteristics of colostrum, although its main function is to support newborns at nutritional level (Henderson et al., 2008; Nommsen-Rivers et al., 2012).

Finally, 2 weeks after the delivery the milk can be considered as mature and its composition tends to be stable over the time, even if slight variations can occur during lactation (Ballard and Morrow, 2013). The main components of human milk are: (i) macronutrients, such as protein, fat and lactose, which concentration depends on the stage of lactation and maternal characteristics; (ii) micronutrients, including vitamins A, B1, B2, B12 and D that vary in human milk in relation to maternal diet and body stores; (iii) growth factor, which are strongly active on the endocrine system, nervous system, vasculature and intestinal tract; (iv) immunological factors, which are essential to defend the newborn from inflammation and infection, and for this reason, the early milk is rich in immune components that can support infants in the first delicate stages of their life; (v) the microbiota, which comprises more than 200 different bacterial species with a pivotal role in the formation of the newborn’s first gut microbiota (Drago et al., 2017).

The aim of the present Mini Review is to highlight the specific and fundamental role of human milk-associated bacteria in modulating and influencing the newborns’ immune system during their life.

## The Milk Microbiota and the Newborn’S Immune System

The specific mechanisms that lead to the formation of the human milk microbiota are still unknown; however, there are different hypothesis that can explain the origin of milk-associated bacteria. Indeed, some microorganisms belonging to the maternal skin or infant’s oral cavity may become an integral component of the milk microbiota by means of a milk flow back into mammary ducts during lactation (Rodríguez, 2014). This mechanism may justify the presence of cutaneous and oral bacteria that are recovered in the milk microbiota, such as *Streptococcus* spp. and *Staphylococcus* spp. (Gao et al., 2007; Grice et al., 2009). Interestingly, human milk contained also a great number of intestinal bacteria, which may spread from the maternal intestinal environment by a mechanism involving dendritic cells (DCs) and CD18^+^ cells (Rodríguez, 2014); these cellular types would be able to capture intestinal microorganisms from the gut lumen and transfer them to lactating mammary glands by means of translocation, which results to be increased during late pregnancy and lactation (Rodríguez, 2014). Consequently, the milk microbiota can shape the initial intestinal microbiome of newborns, together with the maternal intestinal and vaginal microorganisms that are ingested by the neonate during the passage through the birth canal (Houghteling and Walker, 2015). Human milk can stimulate the proliferation of numerous *Bifidobacterium* and *Lactobacillus* strains, the main probiotic microorganisms present in the gut, creating an acidic environmental rich in short chain fatty acids (SCFAs) with a protective and nutritive role at intestinal level (Bode, 2012; Walker and Iyengar, 2015). The constant intake, during lactation, of bacteria contained in the human milk leads to the formation of a transient intestinal microbiota that deeply impacts on the newborn’s development, acting mainly on the maturation of his immune system (Houghteling and Walker, 2015). Indeed, several studies underscored the strict link between the gut microbiota signals, the mucosal host defense and the maturation of immune system, both at intestinal and systemic level (Smith et al., 2007; Sekirov et al., 2008; Walker and Iyengar, 2015). It has been showed that an altered colonization of newborns’ gut may lead to a persistent intestinal dysbiosis and, consequently, to immune-mediated and metabolic diseases during infancy and childhood (Gareau et al., 2010; Johnson and Versalovic, 2012). Moreover, breast-fed newborns have shown to possess a more stable intestinal bacterial population and a well-balanced mucosal immune response if compared to the formula-fed ones (Gronlund et al., 2000; Bezirtzoglou et al., 2011); indeed, a healthy intestinal microbiota can induce specific T cell responses and modulate substrates oxidation, decreasing the impact of autoimmune and allergic diseases not only during childhood but also in adulthood (Guaraldi and Salvatori, 2012; Palma et al., 2012). Finally, breastfeeding has been observed to have a protective role against respiratory and gastrointestinal infections between the ages of 7 and 12 months, leading to a general improvement of symptoms associated to gastrointestinal infections (Duijts et al., 2010).

Intestinal bacteria can also stimulate lymphoid elements and positively influence the maturation of both innate and adaptive immune system, as clearly demonstrated by studying germ-free animals (Cash and Hooper, 2005). It has been shown that in germ-free mice the villus capillaries develop poorly during weaning and remained in this condition also during adulthood, suggesting that the intestinal microbiota is fundamental for intestinal blood vessel to be completely developed (Martin et al., 2010). More interestingly, intestinal bacteria can promote B cell development in Peyer’s Patches and increase the production of mucosal IgA, the main antibody class in secretions that acts as first line of defense (Martin et al., 2010).

Moreover, bacterial surface-expressed or secreted ligands can interact with specific receptors on mucosal immune system and enterocytes leading to a self-limited inflammatory response for preventing pathogen mucosal penetration (Round and Mazmanian, 2009; Walker and Iyengar, 2015).

As underlined by Latuga et al. (2014), all newborns have an immature immune system and the cord blood rich in anti-inflammatory T regulatory cells; furthermore, infants have a high T helper 2 (Th2) that promotes humoral immunity with the production of IL4, IL6 and IL21, thus promoting an increased B cell response and, potentially, a higher allergic sensitization (Latuga et al., 2014). The pivotal role of milk-associated microbiota in influencing the neonates’ immune system is over-emphasized by the cytotoxic function promoted by microbial ligands in breast milk (Donnet-Hughes, 2008). Indeed, *in vitro* stimulation of DCs with lipopolysaccharide can lead to T-cells differentiation, supporting the hypothesis that mature milk may implement the maturation of cytotoxic Th1 cells and improve their activity against infections (M’Rabet et al., 2008). Probably, commensal colonic bacteria may stimulate the release of specific cytokines that create a balanced microenvironment suitable for naive Th0 cells to ripen toward Th1 cell type (Walker and Iyengar, 2015).

*Bacteroides* is a bacterial genus that is very abundant in human colostrum and it may have a main role in the early stages of newborns’ gut colonization, as reported by Mazmanian and Kasper (2006). In particular, the polysaccharide A located on the surface of *Bacteroides fragilis* can interact with Toll receptor 2 on intestinal DCs to stimulate cytokine production which, in turn, favor the proliferation of FOXP3 T cells in the lamina propria. FOXP3 belongs to the forhead transcription factor family bindweed in the expansion of regulatory T cells, thus having a suppressive role in the host’s immune system (Kim, 2009). Therefore, it is clear that a correct stimulation of neonates’ intestinal environment is fundamental for the physiological development of mucosal immune system and tolerance; the latter, in particular, is extremely important to avoid developing allergy or autoimmune diseases (Walker and Iyengar, 2015). Indeed, germ-free animals cannot develop tolerance due to the lack of intestinal bacteria and only the adequate colonization of newborns’ gut can lead to a complete tolerance generation (Karlsson et al., 1999; Olszak et al., 2012).

Consequently, breastfeeding is essential for oral tolerance in newborns, as it is extremely important for the establishment of local and systemic immune tolerance to antigens ingested during lactation (Verhasselt, 2010). Infants are daily exposed to specific antigens, part of which belong directly to the human milk microbiota, and that can translocate across the intestinal barrier, being involved in the presentation by antigen-presenting cells to T lymphocytes. Furthermore, bacteria located in the human milk are fundamental to correctly stimulate the Peyer’s patches, increasing the number of IgA-producing plasma cells in the intestinal environment of newborns (Gross, 2007). Consequently, IgA can trap food antigens favoring their elimination by specific enzymes, avoid the adherence of viruses and microorganisms to intestinal mucosa also counteracting the proliferation of pathogens and exert a direct immunomodulatory activity (Verhasselt, 2010).

Finally, the oral tolerance seems to be actively involved in the prevention of allergic diseases onset in babies, avoiding also the impact of respiratory and gastrointestinal infections during the early stages of their life (Lack, 2008).

Interestingly, some strains contained in the breast milk can stimulate immune responses in both animal and human models and their activity seems to have a moderate suppleness based on the gut environment (Fernández et al., 2013). Some *Lactobacillus* strains can enhance the production of Th1 cytokines as well as TNF-alpha even without inflammatory stimuli and activate NK cells, CD4+ and CD8+ T cells and regulatory T cells.

**Table 1** summarizes the main immunomodulatory activities of human milk microbiota.

**Table 1:** Immunomodulatory activities of the human milk microbiota.

Immunological activity	Reference
Maturation of local and systemic immune system	Houghteling and Walker, 2015
Implementation of mucosal newborns’ defense	Smith et al., 2007; Sekirov et al., 2008
Stimulation of specific cytokines that create a balanced microenvironment	Walker and Iyengar, 2015
Correct development of B cells	Martin et al., 2010
Stimulation of cytotoxic Th1 cells	M’Rabet et al., 2008
Correct balancing between Th1 and Th2 response	Walker and Iyengar, 2015
Development of oral tolerance	Verhasselt, 2010
Stimulation of Peyer’s patches	Gross, 2007

## Microbial Network: New Insights Into Bacterial Ecology

The study of microbial interactions within a bacterial population is of extreme importance to clearly understand the specific role of microbiome. Indeed, microorganisms compete for nutrients, exchange genetic material and metabolites, being responsible of influencing the microbiota composition and the host’s health (Layeghifard et al., 2017). Due to its dynamic nature and high heterogeneity, the microbiota can be considered a complex and variable ecosystem not often well understandable. For this reason, in the last years a novel approach has been developed to study the microbiota, by using graph-theoretical, systems-oriented method able to facilitate the understanding of evolutionary and complex ecological processes (Layeghifard et al., 2017). Bacterial network is becoming essential to study microbial relationships and clarify the impact of various interactions on the host by identifying the main “hubs” that may represent the most influential member in a bacterial community (Layeghifard et al., 2017). Moreover, a central node is thought to have more links with other hubs, having a pivotal role in the stability of the whole microbial network.

Numerous methods to identify network hubs exist, but in the last years there was a great interest in using and apply the Auto-Contractive map (AutoCM). AutoCM system is a fourth-generation unsupervised artificial neural network (ANN) that can outperform numerous unsupervised algorithms (Buscema and Sacco, 2016). The system uses the minimum spanning tree (MST) theory to underline the natural connections between variables (Buscema and Grossi, 2008; Buscema et al., 2008). A MST is a spanning tree of a connected, undirected graph that links all vertices together with the minimal total pondering for its edges. The concept is that as all biological and natural systems are inclined to a minimal energetic condition, the graph delineates the essential biological information of this process. The final goal of this approach is finding out all specific correlations between variables, creating a semantic map of connections in which non-linear associations are maintained. This approach allows to both highlight the relevant nodes of the system and show the network of the main relations among variables. Nodes, also defined as “hubs,” are defined as variables with the highest number of relations in the map (Buscema and Grossi, 2008; Buscema et al., 2008; Buscema and Sacco, 2016).

AutoCM has already been used in the microbiological field to study the bacterial network of human colostrum and mature milk in Italian and Burundian populations, highlighting the main microbial hubs that represent the biological leading structure of the whole network (Drago et al., 2017). This mathematical approach could partially clarify the complexity of human-bacteria mutualism, and above all the role that some specific microorganisms have in the human milk.

**FIGURE 1:**
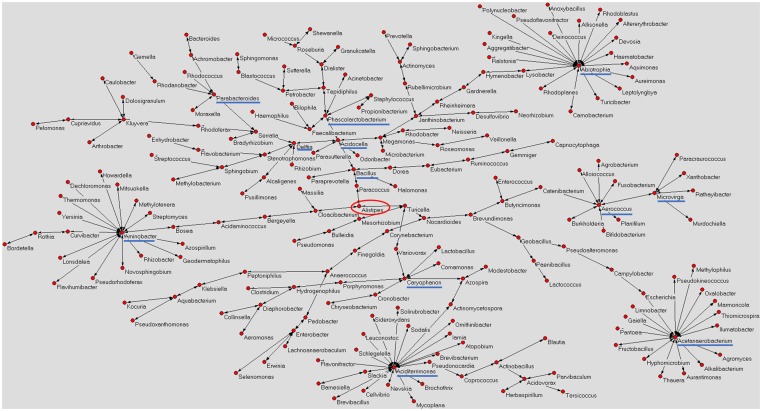
Microbiota network of Italian mature milk. The figure shows the distribution of bacterial population contained in the Italian human mature milk. The network was obtained applying the AutoCM. The main *hubs* of the bacterial network are underlined with a blue line; red circle shows the *central node* of the network (Drago et al., 2017). As authors of the previously published manuscript, we maintain the ownership of copyright as stated in the license-to-publish we signed for the Nature Publishing Group.

**Figure 1** shows a graphical representation of the human mature milk microbiota network obtained using AutoCM. The main bacterial hubs (blue lines) represent those microorganisms that may have a pivotal and leader role in the whole microbiota (Drago et al., 2017).

In the future, the AutoCM may be useful in understanding which microbe is directly involved in the stimulation and manipulation of newborns’ immune system, maybe defining a “immunomodulatory bacterial pattern” with a pivotal role in the development of infant’s immunity.

## Conclusion

The human milk microbiota seems to have a central role in stimulate newborns’ immune system, contributing to create the first transient intestinal microbiota with strong immunomodulatory activities. However, further studies are needed to highlight the direct and strong connection between the human milk microbiota and the stimulation of newborns’ immune system, as to date there are no clear and specific evidence about this association.

Application of network biology will significantly improve our knowledge on bacterial interactions among the milk microbiota, with important applications for eventual targeted modification of bacterial composition, aimed to enhance the abundance of those microorganisms that may be essential not only for the modulation of the infants’ immune system, but also for improving the whole host’s health.

## Author Contributions

LD provided the general concept. LD and EG drafted part of the manuscript. MT and RDG wrote the manuscript. All authors revised and approved the manuscript.

## Conflict of Interest Statement

The authors declare that the research was conducted in the absence of any commercial or financial relationships that could be construed as a potential conflict of interest.
